# Paralogous translation factors target distinct mRNAs to differentially regulate tolerance to oxidative stress in yeast

**DOI:** 10.1093/nar/gkad568

**Published:** 2023-07-14

**Authors:** Joanne Cunningham, Aristeidis P Sfakianos, Paraskevi Kritsiligkou, Christopher J Kershaw, Alan J Whitmarsh, Simon J Hubbard, Mark P Ashe, Chris M Grant

**Affiliations:** Faculty of Biology, Medicine and Health, The University of Manchester, Michael Smith Building, Oxford Road, Manchester M13 9PT, UK; Faculty of Biology, Medicine and Health, The University of Manchester, Michael Smith Building, Oxford Road, Manchester M13 9PT, UK; Faculty of Biology, Medicine and Health, The University of Manchester, Michael Smith Building, Oxford Road, Manchester M13 9PT, UK; Faculty of Biology, Medicine and Health, The University of Manchester, Michael Smith Building, Oxford Road, Manchester M13 9PT, UK; Faculty of Biology, Medicine and Health, The University of Manchester, Michael Smith Building, Oxford Road, Manchester M13 9PT, UK; Faculty of Biology, Medicine and Health, The University of Manchester, Michael Smith Building, Oxford Road, Manchester M13 9PT, UK; Faculty of Biology, Medicine and Health, The University of Manchester, Michael Smith Building, Oxford Road, Manchester M13 9PT, UK; Faculty of Biology, Medicine and Health, The University of Manchester, Michael Smith Building, Oxford Road, Manchester M13 9PT, UK

## Abstract

Translation initiation factor 4G (eIF4G) is an integral component of the eIF4F complex which is key to translation initiation for most eukaryotic mRNAs. Many eIF4G isoforms have been described in diverse eukaryotic organisms but we currently have a poor understanding of their functional roles and whether they regulate translation in an mRNA specific manner. The yeast *Saccharomyces cerevisiae* expresses two eIF4G isoforms, eIF4G1 and eIF4G2, that have previously been considered as functionally redundant with any phenotypic differences arising due to alteration in eIF4G expression levels. Using homogenic strains that express eIF4G1 or eIF4G2 as the sole eIF4G isoforms at comparable expression levels to total eIF4G, we show that eIF4G1 is specifically required to mediate the translational response to oxidative stress. eIF4G1 binds the mRNA cap and remains associated with actively translating ribosomes during oxidative stress conditions and we use quantitative proteomics to show that eIF4G1 promotes oxidative stress-specific proteome changes. eIF4G1, but not eIF4G2, binds the Slf1 LARP protein which appears to mediate the eIF4G1-dependent translational response to oxidative stress. We show similar isoform specific roles for eIF4G in human cells suggesting convergent evolution of multiple eIF4G isoforms offers significant advantages especially where translation must continue under stress conditions.

## Graphical Abstract

**Graphical Abstract:**
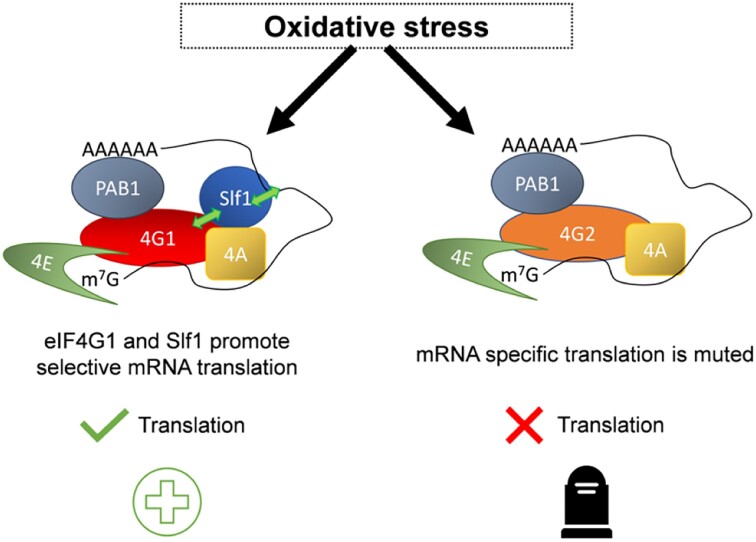

## INTRODUCTION

Translation initiation is the process by which a ribosome is recruited to a messenger RNA (mRNA) transcript to begin protein synthesis. For initiation to occur, the mRNA must first be bound by a series of initiation factors which prepare the transcript and promote recruitment of the ribosome. These stages are crucial, often rate-limiting steps in translation and are subject to extensive regulation, particularly in eukaryotes (1). Translational regulation in response to stress conditions is especially important as cells must be able to make the specific proteins required to survive environmental challenges, whilst not wasting energy making any unnecessary proteins (2). The control of protein synthesis in response to external stimuli is therefore thought to play an important role in the overall regulation of gene expression in eukaryotic cells and some estimates have highlighted a dominant role for translational controls over transcriptional controls (3,4).

For most eukaryotic translation initiation events, the mRNA to be translated must be selected in a process where specific factors interact with the mRNA 5′ cap or 3′ poly(A) tail (5). The eukaryotic initiation factor (eIF)4E interacts with the cap structure (6), whereas the poly(A) binding protein (Pab) binds to the poly(A) tail (7). eIF4G acts as a large scaffold protein which is capable of interacting with both eIF4E and Pab (8). eIF4G also interacts with RNA helicases such as eIF4A, which act to unwind the transcript allowing recruitment of the 43S pre-initiation complex (PIC) and subsequent scanning of the 5′UTR for a start codon (9). The 43S PIC contains the small ribosomal subunit bound by the initiation factors eIF1, eIF1A, eIF3, and the eIF2 Met-tRNA_i_^met^ GTP ternary complex (TC) (1). Critically, eIF4G makes key contacts with eIF3 (in mammals) or eIF5 / eIF1 (in yeast) to recruit the 43S PIC to the mRNA (10–12). The 43S complex then scans the mRNA in a 5′ to 3′ direction until it identifies an AUG start codon in an appropriate sequence context. Start codon recognition by the TC triggers GTP hydrolysis on eIF2 followed by dissociation of eIF2•GDP. Ejection of the majority of the eIFs after start codon recognition and recruitment of the large ribosomal subunit (60S) has been thought to complete the initiation process (1,5). It is generally unclear when and where during scanning and start codon recognition the interactions among eIFs, mRNA, and ribosomal subunits are established and broken (13). For instance, following joining of the 60S subunit, it appears that some eIFs may remain transiently associated with ribosomes through the early elongation and termination phases (14). Both eIF4G and eIF4A are specifically enriched on ribosomes engaged in the translation of short, reinitiation-permissive uORFs (15).

Throughout eukaryotes the mRNA selection process forms a focal point for the regulation of translation initiation. Specific proteins are capable of interacting with the core translation initiation factors to modify their activity. For instance, eIF4E binding proteins (4E-BPs) competitively repress eIF4G binding to eIF4E (16). These proteins target mRNAs either globally or more specifically to reduce the level of translation initiation, often in response to cellular signalling pathways (17). In addition, many of the mRNA selection factors are present as multiple isoforms, which via subtle sequence differences allow fine-tuning of the mRNA selection process. Extreme examples are eIF4E in *Drosophila*, where eight isoforms of the eIF4E gene are present and are thought to direct tissue and developmental specificity to mRNA translation (18). There are also eight isoforms of PAB in *Arabidopsis* (19) and at least ten eIF4A genes have been identified in Tobacco (20). The roles of many of these different proteins are often quite poorly understood.

**Figure 1.**
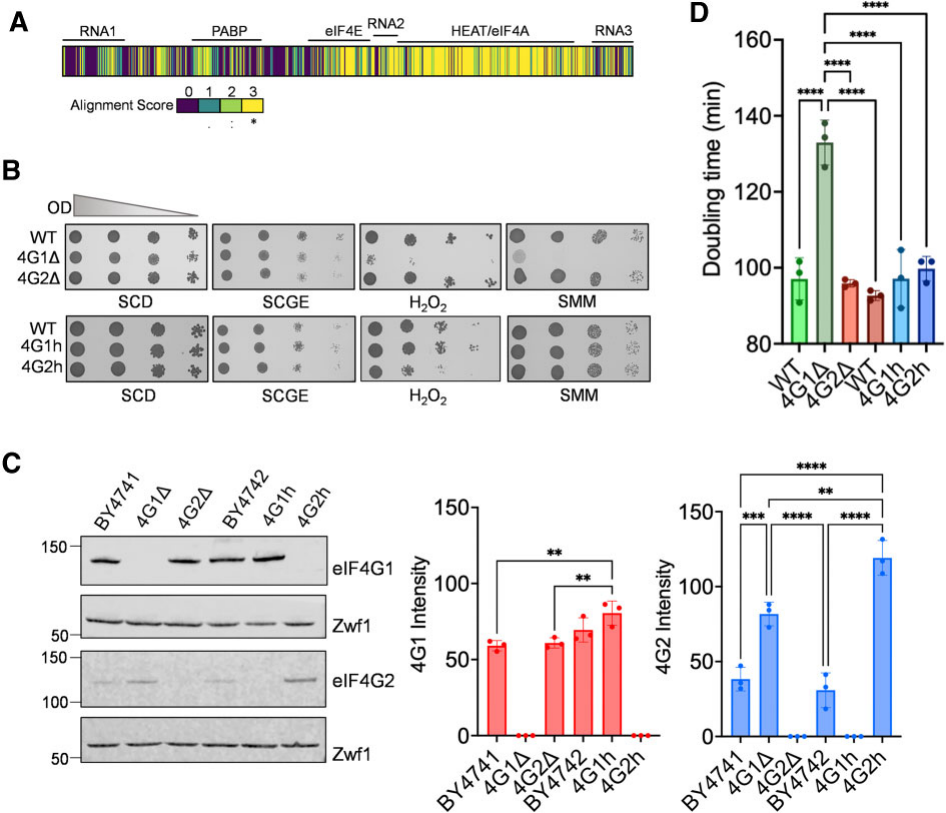
eIF4G1 is required for oxidative stress tolerance. (**A**) Schematic of protein sequence alignment of eIF4G1 and eIF4G2 where yellow sections (scored 3) are conserved residues and purple sections (scored 0) are not similar. Alignment and similarity scores from ClustalOmega (76). (**B**) The wild-type (BY4741) and isogenic eIF4G deletion strains (4G1Δ, 4G2Δ) or wild-type (BY4742) and isogenic homogenic strains (4G1h, 4G2h) were grown to exponential phase and the A_600_ adjusted to 1, 0.1, 0.01 or 0.001 before spotting onto the indicated plates. This included glucose (SCD), glycerol/ethanol, H_2_O_2_ or sulpholmeturon methyl (SMM) plates. (**C**) Western blot analysis of the same strains probed with antibodies that recognise eIF4G1 or eIF4G2 normalized to Zwf1 (glucose 6-phophate dehydrogenase) as a loading control. Data shown are the means of 3 independent biological repeat experiments ± SD. Significance is shown using a one-way ANOVA test. (**D**) Doubling times for the same strains grown on SCD media. Data shown are the means of three independent biological repeat experiments ± SD. Significance is shown using a one-way ANOVA test. **P < 0.01, *** *P* < 0.001, **** *P* < 0.0001.

For the key scaffold molecule eIF4G, there are at least two isoforms in many eukaryotes, with evidence of differential roles for eIF4G domain-containing proteins in humans, flies, and plants, amongst others (21–23). Humans have three eIF4G genes, eIF4G1, eIF4G2 (DAP5) and eIF4G3. eIF4G1 and eIF4G3 are the main eIF4G isoforms, with most homology to the yeast homologues (24,25), whereas DAP5 is only similar to the C-terminal part of the other proteins, and lacks the eIF4E binding domain (26). In *Saccharomyces cerevisiae* there are two isoforms of eIF4G encoded by *TIF4631* and *TIF4632*, referred to as eIF4G1 and eIF4G2 respectively. Similar to human eIF4G, yeast eIF4G is a large protein which directly interacts with Pab, eIF4E, and eIF4A (27–29). It also contains three RNA binding domains which are important for growth and translation (30). Sequence alignment between the two eIF4Gs in yeast shows conservation mostly towards the C-terminus and in the protein binding domains, with variability towards the N-terminal end and the RNA binding domains (Figure 1A). Although the yeast isoforms are similar, they are only ∼50% identical, leaving ample variability that might contribute to isoform-specific functions.

The presence of multiple eIF4G isoforms in diverse eukaryotes is suggestive that these isoforms may have distinct functions that have been independently selected for at various stages of evolution. However, there is little functional evidence for isoform specificity in regulating translation for the different eIF4G isoforms in *Saccharomyces cerevisiae*. The two *S. cerevisiae* eIF4G genes arose from a whole genome duplication event (WGD), which largely defines the post-WGD clade of yeast species (31,32). After this ancestral genome duplication event, one copy of the duplicated genes was lost for most genes, but some duplicated genes were preserved. *S. cerevisiae* has maintained 551 duplicated genes. Intriguingly, many of these duplicated genes are involved in the process of protein synthesis and the two genes have diverged very little.

Yeast deletion mutants lacking individual eIF4G isoforms are viable, although loss of eIF4G1 has been reported to cause slow growth and impair translation initiation (33,34). These phenotypes are thought to derive from the lowered levels of total eIF4G, since eIF4G1 is more highly expressed than eIF4G2. Later studies have used homogenic yeast strains to avoid the problems associated with the slow growth of eIF4G1 mutants. These are strains which express a single eIF4G isoform at similar levels to the total amount of eIF4G present in a wild-type strain and no differences in growth kinetics or translational activity were found in these strains expressing single eIF4G isoforms (34). These results support little or no isoform specificity between the two eIF4G genes.

In this current study we have re-examined this prevailing model, considering whether the yeast eIF4G isoforms are differentially required across a range of conditions, focussing specifically on tolerance to oxidative stress. In contrast to previous data, we show that eIF4G1, but not eIF4G2, promotes tolerance to oxidative stress, highlighting significant differences in the way these isoforms work to regulate gene expression via translational control.

## MATERIALS AND METHODS

### Yeast strains and plasmids

Yeast strain BY4741 (MATa *his3*Δ*1 leu2*Δ*0 met15*Δ*0 ura3*Δ*0*) and its isogenic derivatives *tif4631::KanMX4* and *tif4632::KanMX4* were obtained from the Euroscarf yeast deletion collection (35). The homogenic eIF4G1 (4G1h) and homogenic eIF4G2 (4G2h) strains were made in BY4742 (*MATα his3Δ1 leu2Δ0 lys2Δ0 ura3Δ0*) and have been described previously (34). TAP-tagged strains in the BY4741 background were obtained from Thermo Scientific Open Biosystems (Waltham, MA, USA) apart from the CDC33-TAP strain which was described previously (36). A CRISPR-based approach was used to delete the *SLF1* ORF in the BY4742 wild-type, 4G1h and 4G2h strains using methodology described in (37).

### Growth and stress conditions

Yeast strains were grown at 30°C with shaking at 180 rpm in SCD complete media (0.67% w/v yeast nitrogen base without amino acids, 2% w/v glucose) supplemented with appropriate amino acids and bases (ForMedium, UK). For oxidative stress experiments, H_2_O_2_ was added to exponential phase cells (OD_600_ 0.6–1.0) to a final concentration of 0.4 mM. Stress sensitivity was determined by growing cells to exponential phase in SCD media and spotting serially diluted cultures (*A*_600_ = 1.0, 0.1, 0.01, 0.001) onto SCD agar plates containing various concentrations of hydrogen peroxide or sulpholmeturon methyl (SMM). For growth in the absence of glucose, SCGE contained 3% w/v glycerol and 1% v/v ethanol instead of glucose.

### Cell extract preparation and polysome profiling

Polyribosomal profiling was performed as previously described (38). Briefly, cell cultures were grown at 30°C to an OD_600_ of 0.6–1.0 before fixing translating ribosomes on mRNA by treatment with cycloheximide. Cells were transferred into pre-chilled Falcon tubes containing cycloheximide (at a final concentration of 0.1 mg/mL). Cells were harvested by centrifugation, washed and lysed with acid-washed glass beads in 200 μl polysome lysis buffer (20 mM *N*-2-hydroxyethylpiperazine-N'-2-ethanesulfonic acid (HEPES) pH 7.4, 2 mM magnesium acetate, 100 mM potassium acetate, 0.5 mM dithiothreitol (DTT), 0.4 mM cycloheximide) by vortexing for 20 s, seven times. For high Mg conditions, the protocol was the same but the polysome lysis buffer contained 20 mM Tris pH 7.5, 5 mM magnesium chloride, 140 mM potassium chloride, 0.5 mM dithiothreitol (DTT), 0.4 mM cycloheximide.

Two *A*_260_ units of lysate were layered onto 15–50% sucrose gradients prepared in 12 ml thin-walled open polyallomer tubes (Seton Scientific) and separated by ultracentrifugation in an SW41 Ti rotor (2.5 h at 278 000 × *g*, 4°C). Profiles were generated by continuous *A*_254_ recording using a UA-6 UV/Vis detector and chart recorder (Teledyne ISCO). Profile images were analysed using ImageJ (version 1.52q) (39). Fractions (14 fractions, 0.8–1 ml) were collected manually for western blotting starting from the top of the gradient. Protein was extracted from sucrose gradient fractions by precipitation in 20% trichloroacetic acid (TCA) overnight at –20°C. Precipitated protein was pelleted by centrifugation (15 min at 20 000 × *g*, 4°C) and washed twice with ice-cold acetone.

### Protein and western blot analysis

Protein samples were resolved on NuPAGE 4–12% Bis-Tris gels (Invitrogen) and transferred to nitrocellulose membranes. Blots were visualised using LI-COR fluorescent secondary antibodies and quantified using LI-COR Image Studio (version 5.2). Primary antibodies used were eIF4G1 (40), eIF4G2 (this study), eIF4E (40), Sui2 (41), phospho-Sui2 (Cell Signalling Technologies), Zwf1 (Sigma Aldrich) and Slf1 (Prof. S. Wolin).

### Immunoprecipitation and cap-affinity chromatography

TAP-tagged proteins were immunoprecipitated as previously described (36). Cap affinity chromatography was performed as described in (42).

### Label-free mass spectrometry analysis

Cell extracts were prepared from four biological replicates essentially as described in (43). Protein samples were prepared for MS by adding equal volumes of sample and protein loading buffer (2 × NuPAGE LDS sample buffer [Invitrogen], 715 mM 2-mercaptoethanol) and incubating for 5 min at 95°C. Samples were briefly run on NuPAGE 4–12% Bis-Tris gels (Invitrogen) and then excised from gels. Samples were dehydrated using acetonitrile and centrifuged under vacuum. Dried gel pieces were reduced with 10 mM DTT and alkylated with 55 mM iodoacetamide, then twice washed alternately with 25 mM ammonium bicarbonate and acetonitrile. Gel pieces were dried by vacuum centrifugation and samples digested using trypsin overnight at 37°C.

Liquid chromatography was carried out using an UltiMate 3000 Rapid Separation Binary System (Thermo Fisher Scientific). Peptides were concentrated using an ACQUITY UPLC M-Class Symmetry C18 Trap Column (180 μm inner diameter, 20 mm length [Waters]) and then separated using an ACQUITY UPLC M-Class Peptide BEH C18 Column (75 μm inner diameter, 250 mm length, 1.7 μm particle size [Waters]). A gradient starting with 99% Buffer A (0.1% formic acid in water) and 1% Buffer B (0.1% formic acid in acetonitrile) and increasing to 75% Buffer A and 25% Buffer B was used to separate the peptides over 45 min at a flow rate of 200 nl/min. Label-free tandem MS was performed using an Orbitrap Elite Hybrid Ion Trap-Orbitrap Mass Spectrometer (Thermo Fisher Scientific). Peptides were selected for fragmentation and MS2 analysis automatically by data-dependent analysis.

Raw MS data were processed using MaxQuant version 1.6.17.0 (44). A peptide mass tolerance of 20 ppm was used for the first search, 4.5 ppm for the main search, and 0.5 Da for the MS/ MS fragment ions. The ‘match between runs’ feature was used. The peak list was searched against the Uniprot *Saccharomyces cerevisiae* database (accessed 10 February 2017) using the built-in Andromeda search engine (45). Data from the ‘proteinGroups’ file produced by MaxQuant were imported into R (RStudio 2021.09.2 + 382). One biological replicate from the ‘4G2h Unstressed’ condition was identified as an outlier and omitted from the analysis. Proteins identified as potential contaminants, reverse proteins, and only identified by site were filtered out. Proteins without a gene name were filtered out. Proteins with only one razor and unique peptide were filtered out. Normalisation was performed using the limma package (46). The *normalizeQuantiles* function was used to normalise the LFQ intensities by the mean of each quantile yielding the same empirical distribution for each sample. All protein intensities (‘LFQ.intensity’ columns) were log_2_-transformed and filtered by >2 valid values in a condition using Perseus (version 1.6.15.0) (47). The mass spectrometry proteomics data have been deposited to the ProteomeXchange Consortium via the PRIDE (48) partner repository with the dataset identifier PXD041682.

### Bioinformatic analyses

GO analysis was performed using the clusterProfiler package (version 3.18.1) (49). The *compareCluster* function was used to compare GO term enrichments between proteins significantly different in pairwise comparisons (parameters: organism database = *S. cerevisiae* (org.Sc.sgd.db), *P*-value cutoff = 0.05, function = ‘enrichGO’). Following overrepresentation analysis, the *simplify* function was used to remove overlapping GO terms. GOSlim analysis using PANTHER (Version 17.0) was used to generate simplified GO term enrichment categories between the same lists (50,51).

### HeLa cell culture and SiRNA

HeLa cells (from ATCC) were grown at 37°C and 5% CO_2_ in Dulbecco's modified Eagle's medium (D6429, Gibco® Life Technologies) with the addition of 10% foetal bovine serum (FBS) (S181H, Biowest) and 1% penicillin–streptomycin solution (P4333, Sigma). 100 000 cells/well were platted in 6-well plates for both immunoblotting and immunofluorescence assays. For immunofluorescence experiments, cells were grown on cover slips. Knock-down of eIF4G1 and eIF4G3 was achieved either using a control siRNA (D001810-01-05) or by using an eIF4G1 or eIF4G3 ON-TARGET plus Human siRNA smartpool provided by Horizon and LipofectamineTM RNAiMAX Transfection Reagent (13778075, ThermoFisher Scientific) according to the manufacturer's protocol. Confirmation of mRNA knock-down was achieved via qPCR using TaqMANTM probes against eIF4G1 mRNA (Hs00191933, ThermoFisher Scientific) and eIF4G3 (Hs01554185_m1, ThermoFisher Scientific). For both immunofluorescence and western blot experiments, eIF4G1 and eIF4G3 were probed using anti-eIF4G1 antibody (ab2609, Abcam) and anti-eIF4G3 antibody (PA5-31101, ThermoFisher Scientific). MitoTracker Red FM (Invitrogen) was used for mitochondria staining. Cell viability was measured using crystal violet reagent (C0775, MERCK).

## RESULTS

### eIF4G1 is required for oxidative stress tolerance

The presence of two diverged eIF4G genes in the yeast *Saccharomyces cerevisiae* provides a flexible system for studying the role of paralogous translation factor genes. Previous work has suggested that the underlying rationale for maintaining two eIF4G genes relates to the dosage of eIF4G. Such a hypothesis is difficult to link with the high degree of divergence in protein sequence between eIF4G1 and eIF4G2, and the fact that over 85% of genes have been lost after the genome duplication event in *S. cerevisiae* (52). Previous work on the sub-functionalization of duplicated genes in yeast has posited that differential functionality is often associated with stress response pathways (53,54). Therefore, to explore whether there are any functional differences in the requirement for the duplicated eIF4G genes, the stress sensitivity of different eIF4G mutants was examined.

Stress conditions were selected that are known to cause a rapid attenuation of protein synthesis including oxidative stress, amino acid starvation and glucose depletion (55–57). Strains were grown to exponential phase and serially diluted before spotting onto plates containing hydrogen peroxide to cause oxidative stress, sulpholmeturon methyl (SMM) to cause an amino acid starvation (isoleucine/valine starvation) or plates containing glycerol/ ethanol instead of glucose (SCGE). We initially used deletion strains where either *TIF4631* encoding eIF4G1 (denoted 4G1Δ) or *TIF4632* encoding eIF4G2 (denoted 4G2Δ) was deleted. The 4G1Δ strain was strongly sensitive to oxidative stress and amino starvation compared with the wild-type and 4G2Δ strains (Figure 1B). No differences in sensitivity to growth on SCGE were observed.

Given previous reports that phenotypes observed in the 4G1Δ strain might arise due to lowering the eIF4G dose rather than through any isoform-specific effects, it was important to measure the cellular concentrations of eIF4G1 and eIF4G2 in the deletion strains (34). Using isoform-specific antibodies, we found that a similar concentration of eIF4G1 was present in both the 4G2Δ and wild-type strains (Figure 1C). In contrast, the cellular concentration of eIF4G2 increased approximately two-fold in the 4G1Δ strain presumably due to the previously reported compensatory transcriptional increase in *TIF4632* expression observed in 4G1Δ strains (34). Thus, even though eIF4G2 concentrations are significantly increased in the 4G1Δ strain, it is not sufficient to promote oxidative stress tolerance, suggesting that differences in stress tolerance may arise due to functional differences between the two proteins.

We next examined the stress sensitivity of homogenic strains which are strains where the coding region of eIF4G1 is replaced with the coding region of eIF4G2 in one strain (4G2h), and in another strain the coding region of eIF4G2 is replaced with eIF4G1 (4G1h) (34). The homogenic eIF4G1 (4G1h) strain expressed eIF4G1 at comparable levels to eIF4G1 present in the wild-type strain (Figure 1C). The cellular concentrations of eIF4G2 were again significantly increased in the 4G2h strain which only contains eIF4G2 and lacks any eIF4G1. Analysis of stress sensitivity for the 4G1h and 4G2h strains revealed that neither strain was affected in growth on SMM or SCGE relative to the wild type-strain, whereas the 4G2h homogenic strain was sensitive to oxidative stress (Figure 1B). It is unclear why the 4G1Δ strain is sensitive to SMM, whereas the 4G2h strain is resistant. It may be related to the different amino acid auxotrophies present in the deletion (BY4741 – *met15*) and homogenic (BY4742 – *Lys2*) strains since SMM is an inhibitor of acetolactate synthase (ALS), a branched-chain amino acid biosynthetic enzyme (58). However, the consistent sensitivity of both the 4G1Δ and 4G2h strains to hydrogen peroxide indicates that eIF4G1 is required for oxidant tolerance, and the rest of this study has focused on the role of eIF4G in oxidative stress tolerance.

Comparison of growth rates for the various strains indicated that the 4G1Δ strain is slow growing, whereas the homogenic strains grow at wild-type rates (Figure 1D). The fact that the 4G1Δ strain is slow growing compared with the 4G2h strain presumably reflects a threshold affect in the requirement for eIF4G2 cellular concentrations to support the growth of a strain lacking eIF4G1. Subsequent studies have therefore focussed on the homogenic eIF4G strains to avoid any complications that might arise due to differences in growth rate.

### eIF4G1 is required to remodel the proteome in response to oxidative stress

We reasoned that the differential requirement for eIF4G1 to promote tolerance to oxidative stress might arise due to differences in translation and protein production driven by the presence of the different eIF4G isoforms. To examine any oxidative stress induced proteome changes, whole cell extracts were prepared and proteome changes analysed using a label-free quantitative mass spectrometry (LC–MS) approach comparing total cell extract proteomes (59,60). For these experiments, the wild-type and homogenic strains were grown to exponential phase before treatment with 0.4 mM H_2_O_2_ for 1 h.

**Figure 2.**
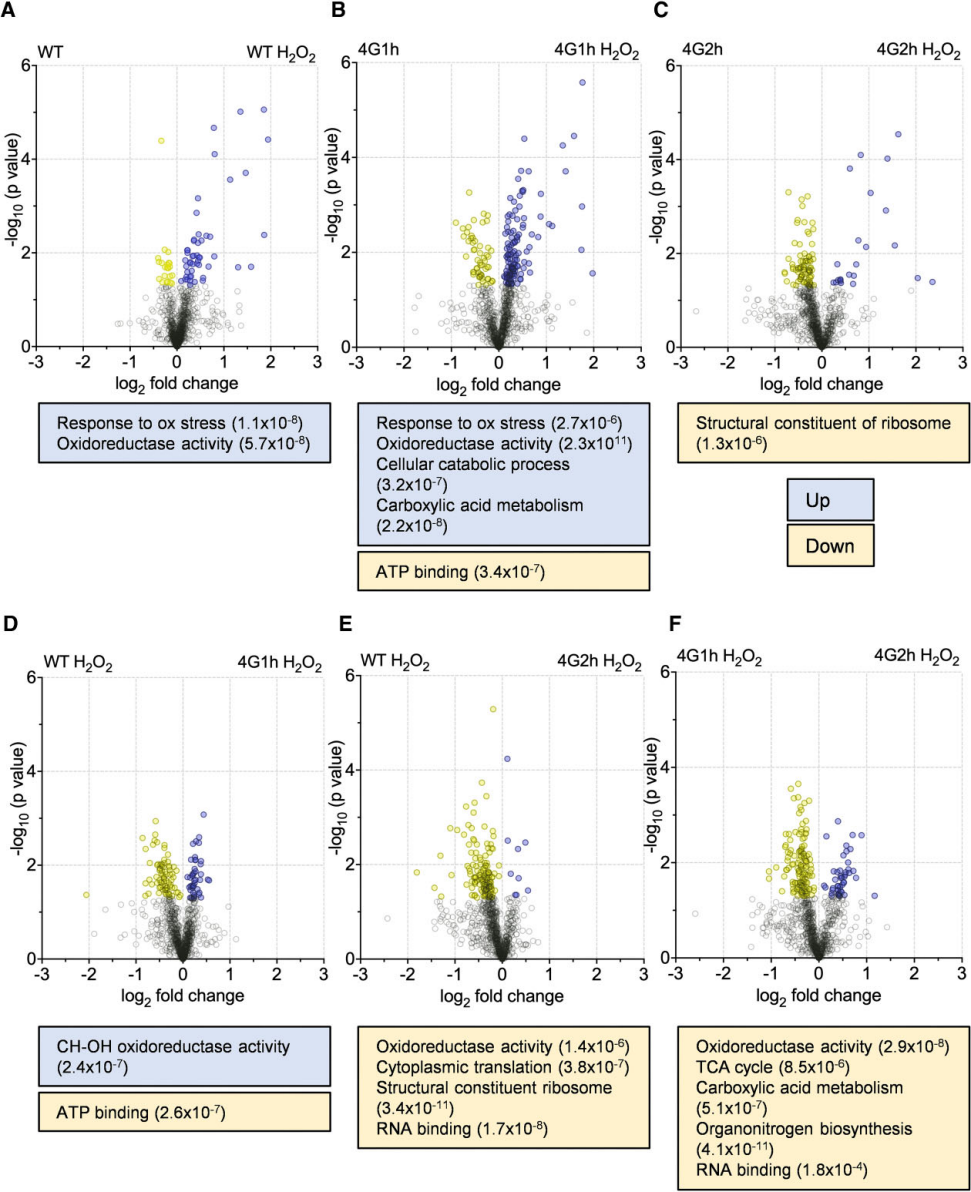
eIF4G1 is required to remodel the proteome in response to oxidative stress. (**A–C**) Volcano plots are shown comparing the values for log_2_(fold change) and -log_10_(*p* value) values for proteins identified in the wild-type and homogenic strains in the presence or absence of oxidative stress conditions. (**D–F**) Pairwise comparisons are shown for the indicated strains in the presence of hydrogen peroxide. Proteins which are significantly increased (*P* < 0.05) are coloured blue and proteins which are significantly decreased are coloured yellow (*P* < 0.05) and are listed in Supplementary Table S1. GOSlim enrichments are shown for significantly different proteins in each comparison.

The differences in protein abundance between the homogenic strains and oxidative stress conditions were first assessed using volcano plots where the log_2_(fold change) and –log_10_(*P* value) for different pairwise comparisons were plotted, and proteins that were significantly different across the comparison highlighted (Figure 2). Following one hour of oxidative stress treatment, there were significant changes to all the strains’ proteomes. For the wild-type and 4G1h strains there was a skew towards higher protein abundance under oxidative stress conditions (Figure 2A, B). However, for the 4G2h strain higher protein levels were seen pre-stress and fewer proteins increased in level after oxidative stress conditions (Figure 2C). These differences are also highlighted by the lists of significantly upregulated proteins for each strain following oxidative stress, where the wild-type strain had 51 proteins which were significantly more abundant after stress, the 4G1h strain had 118, but the 4G2h strain only had 22. This indicates that the 4G2h response to stress may be more muted in comparison to the wild-type or 4G1h strains, potentially explaining the observed differences in sensitivity to H_2_O_2_. The deficiencies in the 4G2h oxidative stress response were further emphasised by the gene ontology (GO) analysis. For instance, enrichment of the biological process category ‘Cellular response to oxidative stress’ and the molecular function category ‘Oxidoreductase activity’ were only found in the wild-type and 4G1h strain samples after H_2_O_2_ treatment, but not in the 4G2h H_2_O_2_ condition (Figure 2A–C).

To further compare the oxidative stress proteomes of the homogenic strains, each of the strains’ proteomes following oxidative stress were subject to pairwise comparisons. Strikingly, while the wild-type and 4G1h strains showed very similar proteomes following oxidative stress, the 4G2h strain had significantly lower abundances for many proteins when compared to both the wild-type and 4G1h strains (133 and 127 respectively) (Figure 2D–F). Conversely, there were only nine proteins with a significantly *higher* abundance in the 4G2h strain following oxidative stress compared with the wild-type strain following oxidative stress (Figure 2F). These data illustrate that while the WT and 4G1h strains both express proteins to similar levels of abundance, a substantial number of proteins are present at lower levels in the 4G2h strain under the same condition. Gene ontology analysis again revealed an enrichment for the molecular function category ‘Oxidoreductase activity’ in the wild-type and 4G1h hydrogen peroxide proteomes compared with the 4G2h hydrogen peroxide proteome (Figure 2D–F).

### The translational response to oxidative stress is different in strains containing eIF4G1 or eIF4G2 alone

**Figure 3.**
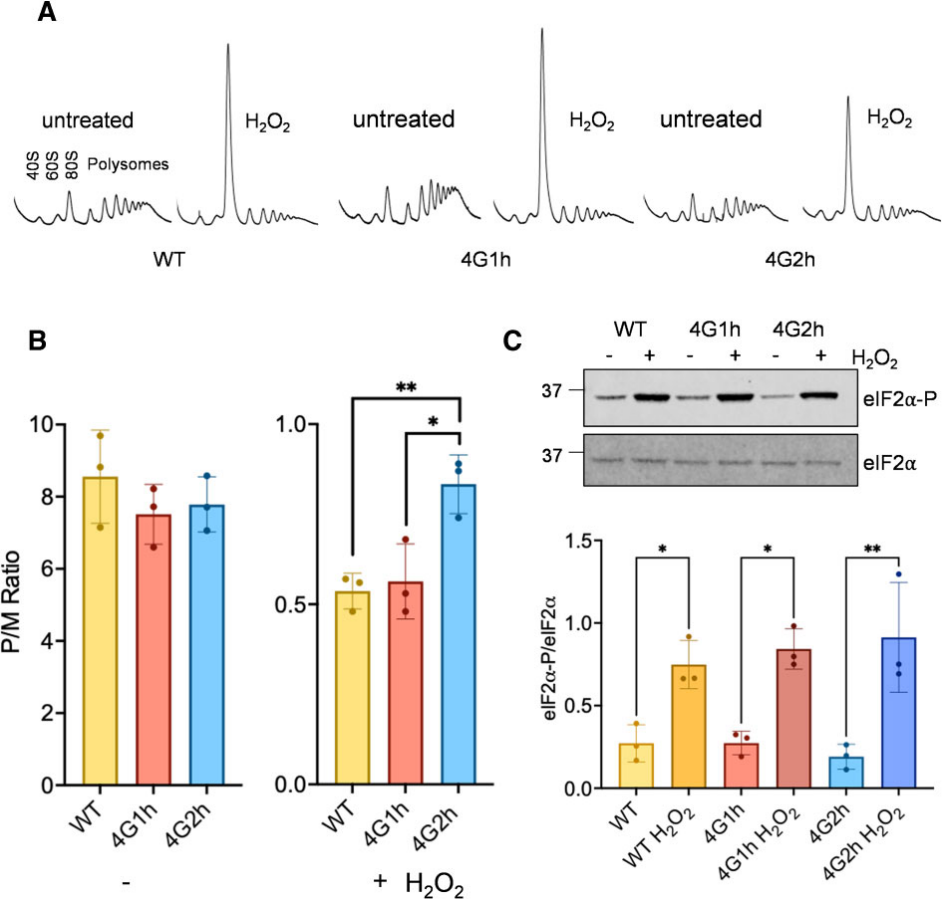
The translational response to oxidative stress is different in strains containing eIF4G1 or eIF4G2 alone. (**A**) Representative polyribosome traces are shown for the wild-type and homogenic strains before or after treatment with 0.4 mM H_2_O_2_ for 15 min. The peaks that contain the small ribosomal subunit (40S), the large ribosomal subunit (60S), and both subunits (80S) are indicated along with the polysome peaks generated by 2, 3, 4, 5, etc., 80S ribosomes on a single mRNA. (**B**) Quantification of polysome:monosome ratios is shown from triplicate traces determined by the ratio between the area under the monosome to the polysome peaks. Error bars denote standard deviation and significance is shown comparing unstressed or stressed ratios using a one way ANOVA test. (**C**) Western blot analysis of eIF2α (Sui2) and eIF2α-P using the same strains and conditions as for panels A and B. Quantification is shown from triplicate experiments comparing phosphorylation in the presence or absence of hydrogen peroxide for each strain using one-way ANOVA. *P < 0.05, **P < 0.01.

Since the presence of different eIF4G isoforms caused alterations in the proteome and eIF4G is an essential translation factor (33), we next addressed whether eIF4G1 or eIF4G2 affect the translational response to oxidative stress. Translational activity was analysed by examining the sedimentation of ribosomes within a sucrose density gradient during non-stress conditions and in response to hydrogen peroxide. During unstressed growth conditions, similar polysome profiles were observed in the wild-type, 4G1h and 4G2h strains (Figure 3A), similar to previous studies (34). Most ribosomes are in the polysome fractions and so are actively engaged in translating mRNA. Therefore, the translational activity, defined as the polysome to monosome (P/M) ratio was high and comparable in all three strains (Figure 3B).

**Figure 4.**
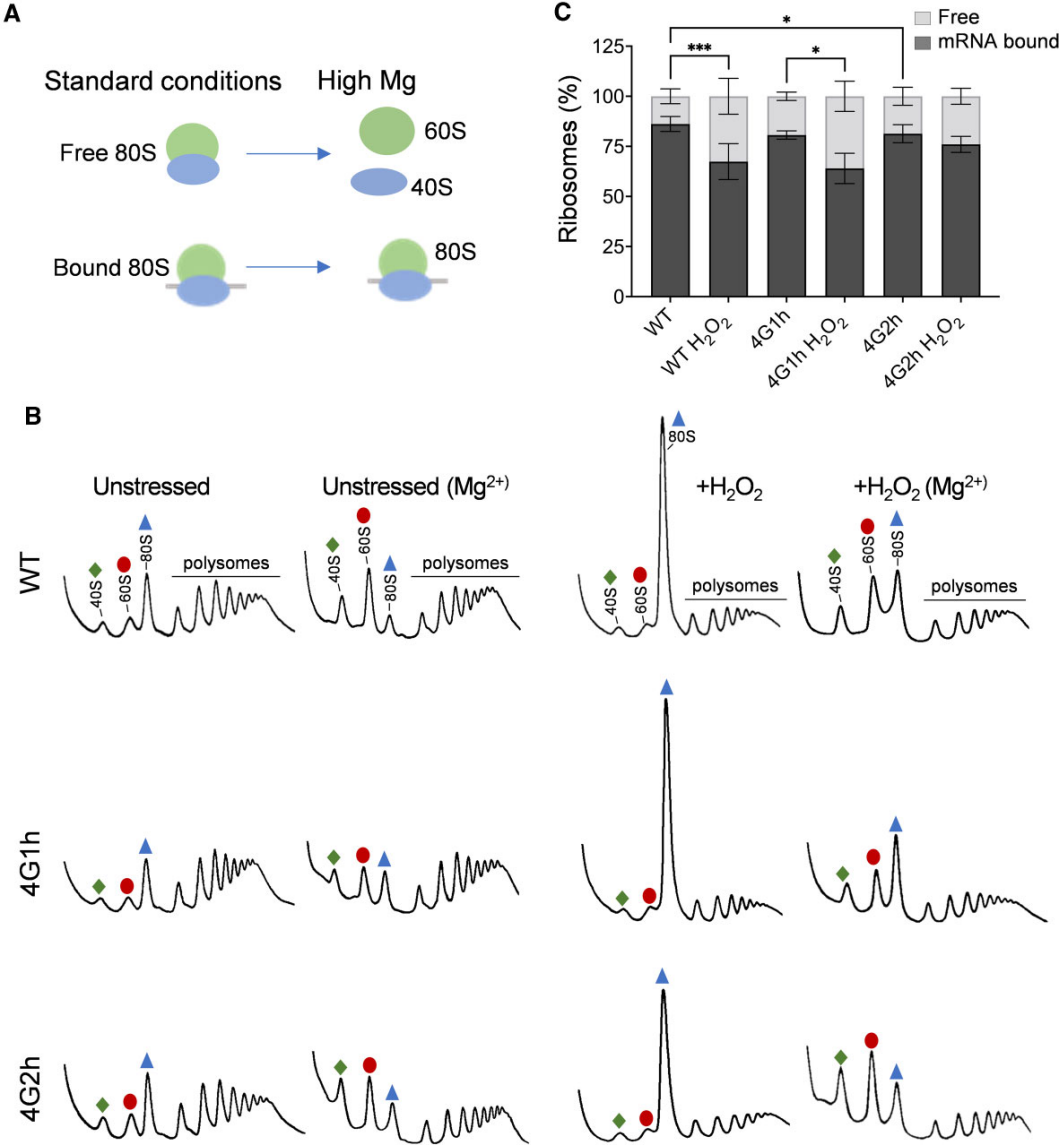
The proportion of mRNA-bound versus free 80S ribosomes increases in eIF4G1 containing strains but not in eIF4G2-containing strains strain following oxidative stress conditions. (**A**) Schematic showing the effects of high magnesium concentrations in polysome buffers on either free or mRNA bound 80S ribosomes. Free 80S subunits dissociate to their 40S and 60S constituent parts whereas mRNA bound 80S subunits are stabilised by their mRNA interaction and remain as 80S monosomes. (**B**) Representative polyribosome traces are shown in the presence or absence of high magnesium for the wild-type and homogenic strains before or after treatment with 0.4 mM H_2_O_2_ for 15 minutes. Green diamonds (40S), red ovals (60S) and blue triangles (80S) denote ribosomes and subunits. (**C**) Quantification of the area of the 40S and 60S peaks (free) and the 80S and polysomes (bound) as a proportion of the total trace, where the free fraction represents ribosomal subunits separated by magnesium and the bound fraction represents ribosomes stabilised by mRNA. Significance is shown using a one-way ANOVA test for the bound fraction; *P < 0.05, ***P < 0.001.

It is well known that stress conditions such as oxidative stress cause a marked inhibition of translation initiation promoted by Gcn2-dependent phosphorylation of eIF2α (56). On polysome gradients, such an inhibition of translation initiation is observed as an increase in the 80S monosome peak along with reduced levels of actively translating polysomes. This is because initiation is the rate limiting step in translation and continued elongation causes a characteristic ‘run-off’ of polysomes resulting in the increased 80S peak (55,61). Accordingly, in keeping with previous studies (56), we found that in the wild-type strain after a 15 minutes exposure to 0.4 mM H_2_O_2_ significant polysome run-off was evident (Figure 3A). This result is characteristic of an inhibition of translation initiation and can be measured from the decreased polysome to monosome (P/M) ratio (Figure 3B). A very similar scale of polysome run-off and decrease in the P/M ratio was also observed in the 4G1h strain. Interestingly however, the P/M ratio observed in the 4G2h strain in response to hydrogen peroxide treatment was significantly higher than for the wild-type (*P* < 0.01) and 4G1h (*P < 0.05) strains (Figure 3A and B). This result suggests that the inhibition of translation initiation caused by oxidative stress may not be so pronounced for the 4G2h strain and the mechanism underlying this finding is further explored in Figure 4. We further verified this result by examining translational activity in the 4G1Δ and 4G2Δ strains exposed to increasing concentrations of hydrogen peroxide. A comparable decrease in the P/M ratio was observed in response to hydrogen peroxide treatment in the wild-type and 4G2Δ strains (Supplementary Figure S1). In contrast, a less dramatic decrease in the P/M ratio was observed in the 4G1Δ strain supporting the idea that eIF4G1 is required for the translational inhibition caused by oxidative stress (Supplementary Figure S1).

Exposure to oxidative stress is known to inhibit translation initiation to cause polysome run-off via the Gcn2-dependent phosphorylation of eIF2α (56). Given the difference in P/M ratios observed between the 4G1h and 4G2h strains after exposure to oxidative stress, we examined whether there are any differences in hydrogen peroxide-induced phosphorylation of eIF2α. This analysis revealed similar increases in eIF2α phosphorylation in response to hydrogen peroxide treatment in the wild-type, 4G1h and 4G2h strains (Figure 3C). This indicates that the difference in 80S monosome accumulation observed in the 4G2h strain does not arise due to any differences in Gcn2-dependent phosphorylation of eIF2α.

### The 4G2h strain is defective in translation initiation following oxidative stress

Oxidative stress is known to inhibit protein synthesis at multiple levels including inhibition at the initiation and elongation phases of translation (56). The decreased 80S monosome peak observed in response to oxidative stress in the 4G2h strain compared with the 4G1h strain might therefore arise due to a more severe elongation block preventing polysome run-off and the accumulation of free 80S ribosomes, or alternatively, it may be due to an initiation block preventing 80S ribosomes from being assembled on mRNAs. The ribosomes that are detected in the 80S monosome peak on standard sucrose density gradients represent both mRNA-free, and therefore translationally inactive 80S ribosomes, or 80S ribosomes that are bound to mRNAs. The 80S ribosomes that are bound to mRNAs are more stable than free 80S ribosomes and high magnesium concentrations can be used in sucrose density gradients to differentially dissociate free 80S ribosomes into their constituent 40S and 60S ribosomal subunits (Figure 4A) (62).

As anticipated, we observed dissociation of the 80S monosome peaks into their constituent 40S and 60S ribosomal subunits for all strains analysed using high magnesium sucrose density gradients (Figure 4B). Quantification of the 80S monosome peaks remaining in the wild-type, 4G1h and 4G2h strains indicated that similar proportions of mRNA bound 80S ribosomes are present in all three strains during non-stress conditions (Figure 4C). In contrast, similar quantitation of mRNA bound 80S ribosomes after hydrogen peroxide treatment revealed a difference for the 4G2h strain. In contrast to the WT and 4G1h strains where the proportion of the 80S ribosomal peak made up of mRNA bound ribosomes increases after stress, for the 4G2h strain there was no increase in the proportion of mRNA-bound versus free 80S ribosomes after oxidative stress (Figure 4C). This difference may explain the reduced height of the 80S monosome peak in the 4G2h strain after stress (Figure 3A and B). This suggests that despite eIF2 being phosphorylated to a similar extent in response to oxidative stress, fewer new initiation events occur in the 4G2h strain during oxidative stress conditions.

### eIF4G1 remains associated with actively translating polysomes following oxidative stress

**Figure 5.**
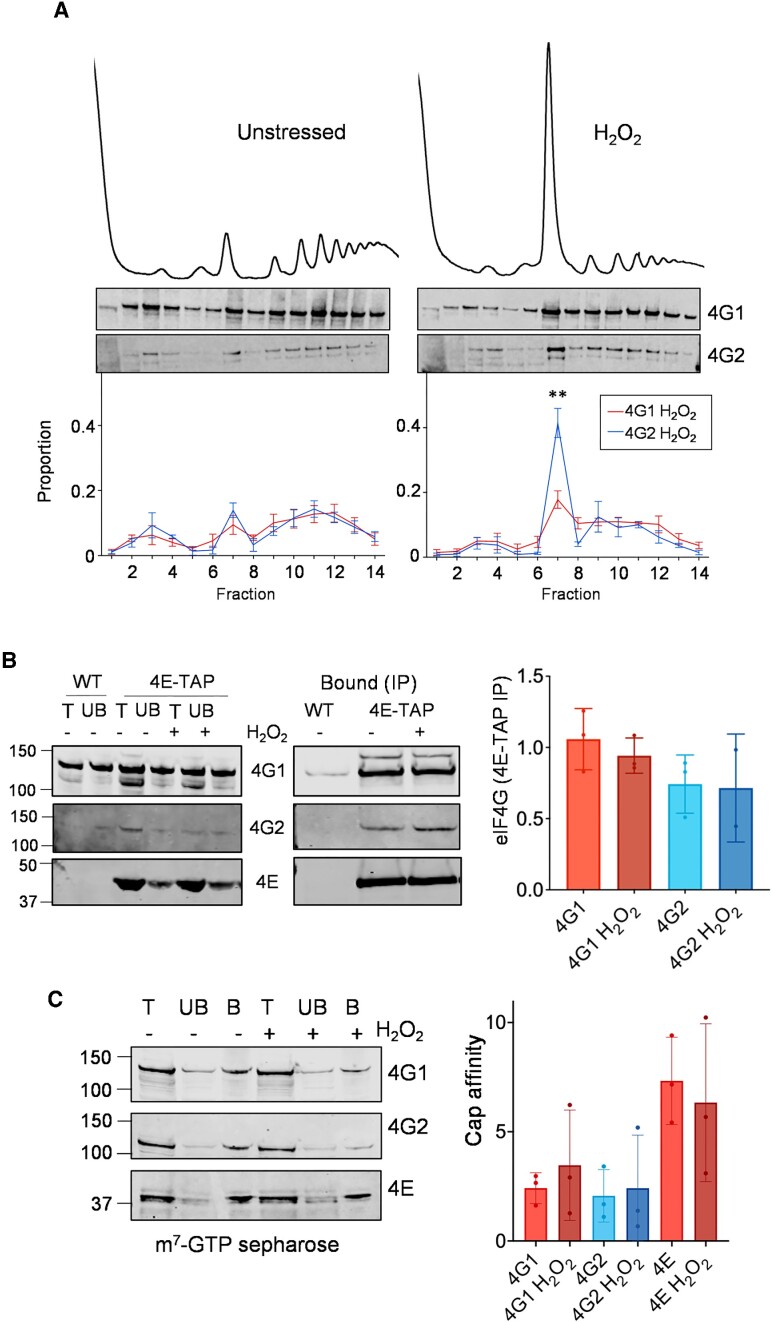
eIF4G1 is associated with actively translating mRNAs following oxidative stress conditions. (**A**) Polyribosome profiles are shown for wild-type strain before or after hydrogen peroxide treatment for 15 min. Sucrose density gradients were fractionated and proteins visualised by Western blotting using antibodies specific for eIF4G1 or eIF4G2. The intensities of eIF4G1 and eIF4G2 bands were quantified and are shown as a percentage of the total cumulative intensity across each polyribosome trace. Quantification is shown from triplicate experiments (pairwise *t*-test, **P < 0.001) (**B**) eIF4E-TAP was immunoprecipitated from an exponential phase whole cell extract either unstressed or treated with 0.4 mM H_2_O_2_ for 15 min. The total (T), unbound (UB) and bound (IP) fractions were analysed by western blotting and membranes probed with antibodies for eIF4E, eIF4G1 and eIF4G2. Quantification is shown for the normalised ratio of eIF4G: eIF4E from triplicate experiments. No significant differences were found using one-way ANOVA. (**C**) A whole cell extracts were prepared from the wild-type strain either unstressed or treated with 0.4 mM H_2_O_2_ for 15 minutes and incubated with m^7^GTP-sepharose. Total (T), unbound (UB), and bound (B) fractions were analysed by Western blotting. Membranes were probed with antibodies for eIF4G1, eIF4G2 and eIF4E. Quantification is shown for bound factions as a percentage of totals. No significant differences were found using one-way ANOVA.

The above data suggest that in the presence of eIF4G1, translation initiation persists at some small level to produce mRNA associated 80S ribosomes under oxidative stress conditions. To explore whether eIF4G1 remains associated with translated mRNAs after stress, the distribution of both eIF4G1 and eIF4G2 in sucrose density gradients was examined. Analysis of the wild-type strain grown under non-stressed conditions revealed that eIF4G1 and eIF4G2 show a similar pattern of distribution with enrichment in the 40S and 80S fractions along with association with ribosomes in the heavy actively translating polysome fractions (Figure 5A). The association of eIF4G1 across a polysome gradient was only subtly altered following oxidative stress conditions. In contrast, a significant proportion of eIF4G2 was shifted from the heavy polysome fractions to the 80S monosome fraction following exposure to hydrogen peroxide (Figure 5A). This means that a lower proportion of eIF4G2 is associated with polysomes compared with eIF4G1 during oxidative stress conditions. The continued association of eIF4G1 with actively translating ribosomes may explain the role of eIF4G1 in promoting translation during oxidative stress conditions.

### No differences in eIF4E association or mRNA-cap binding are detected for eIF4G1 or eIF4G2 following oxidative stress conditions

One possibility for the translation that persists after oxidative stress, is that eIF4G1 is part of a mechanism that favours the selection of stress responsive mRNAs for translation. A major function of eIF4Gs in translation is that together with eIF4E they interact with the mRNA cap. So a key question is whether the eIF4E binding and cap interaction are maintained for eIF4G1 and eIF4G2 after stress. We used two different approaches to examine these interactions. We first compared the interaction between eIF4E and eIF4G during normal and oxidative stress conditions. For this experiment we used an eIF4E-TAP strain and performed TAP affinity purifications and Western blotting to examine the interaction between eIF4E and eIF4G1 or eIF4G2. As expected, eIF4E immunoprecipitated a fraction of both eIF4G1 and eIF4G2 (Figure 5B). However, no differences were observed in the interactions between eIF4E and eIF4G1 or eIF4G2 during normal or oxidative stress conditions.

As a second approach, we used Cap affinity chromatography to assess the ability of eIF4G to bind the mRNA cap structure with eIF4E. Both eIF4G1 and eIF4G2 were found to interact with the mRNA cap although the levels bound across these experiments were quite variable. This analysis revealed that the proportion of eIF4G1, eIF4G2 or eIF4E bound to the cap structure did not change following oxidative stress compared to the unstressed condition (Figure 5C). Differences in eIF4E or mRNA cap binding do not therefore appear to explain the different requirements for eIF4G1 or eIF4G2 during oxidative stress conditions.

### The Slf1 la-related protein (LARP) is required for the eIF4G1-mediated response to oxidative stress

Taken together, our data so far suggest that the continued association of eIF4G1 with actively translating mRNAs may mediate the translational regulation of mRNAs that are required for stress adaptation. This raises the question as to how these mRNAs are translated during oxidative stress conditions that cause a global inhibition of translation via increased eIF2α phosphorylation. A large number of RNA-binding proteins (RBPs) are known to interact with translation factors and/or the ribosome to either promote or inhibit mRNA translation during different growth and stress conditions (63). One key example is the yeast La related protein Slf1, which is a key activator of translation during oxidative stress conditions and has also been shown to remain associated with polysomes during ROS exposure (64). Additionally, similar to the increased P:M ratio observed in the 4G2h strain in response to oxidative stress (Figure 3A), the P:M ratio is also increased in an *slf1* mutant compared with a wild-type strain following exposure to the same concentration of hydrogen peroxide (64). We therefore tested the interaction between eIF4G and Slf1, and whether Slf1 is required to promote eIF4G1-dependent oxidant tolerance.

**Figure 6.**
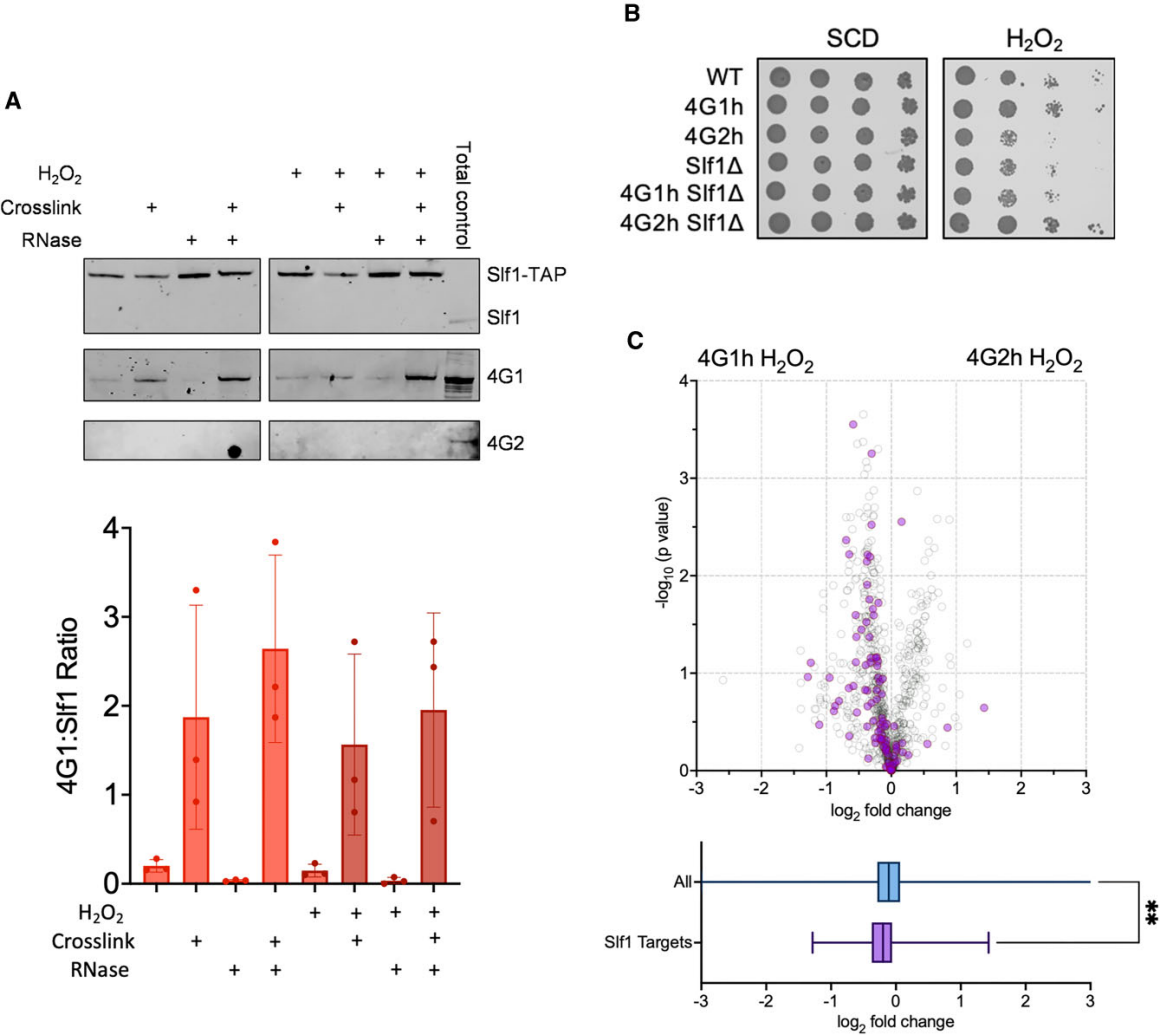
Slf1 is required for the eIF4G1-mediated response to oxidative stress. (**A**) Slf1-TAP was immunoprecipitated from an exponential phase whole cell extract either unstressed or treated with 0.4 mM H_2_O_2_ for 15 minutes, formaldehyde, or RNase I. The bound (IP) fractions were analysed by Western blotting and membranes probed with antibodies for Slf1, eIF4G1 and eIF4G2. (**B**) The wild-type (BY4742), homogenic strains (4G1h, 4G2h), and strains deleted for *SLF1* (*slf1*, 4G1h *slf1*, 4G2h *slf1*) were grown to exponential phase and the A_600_ adjusted to 1, 0.1, 0.01 or 0.001 before spotting onto the indicated plates. (**C**) Volcano plot of the log_2_ fold change and –log_10_*P* value of the pairwise comparisons between the homogenic strains in oxidative stress conditions with Slf1 mRNA targets highlighted in purple. Box and whisker plots show the average fold changes for all proteins and Slf1 mRNA targets.

Previous studies found that Slf1 immunoprecipitated a fraction of key closed-loop proteins consistent with the idea that Slf1 remains associated with actively translating mRNAs during oxidative stress conditions (64). This included eIF4G1, but the interaction with eIF4G2 was not examined. We therefore repeated this immunoprecipitation using Slf1-TAP and the resulting western blot was probed using antibodies specific for eIF4G1 and eIF4G2. Slf1 was found to precipitate a fraction of eIF4G1 during normal and oxidative stress conditions as expected (Figure 6A). In contrast we could not detect any interaction between Slf1 and eIF4G2.

The previous study found that RNase I treatment diminished the co-immunoprecipitation of Slf1 and eIFG1 which was taken as evidence that this interaction might be mRNA mediated (64). We first confirmed that the interaction between Slf1 and eIF4G1 is decreased by treatment with RNase I (Figure 6A). We reasoned that eIF4G1 and Slf1 maybe in close proximity on mRNAs and so we used formaldehyde cross-linking to stabilise protein and RNA interactions. This increased the amount of eIF4G1 that was immunoprecipitated by Slf1, but again we could not detect any precipitation of eIF4G2 (Figure 6A). Formaldehyde cross-linking was also found to prevent the decreased eIF4G1–Slf1 interaction observed in response to RNase I treatment (Figure 6A). Taken together these data indicate that not only do Slf1 and eIF4G1 appear to interact in an RNA-dependent manner, but they must do so in close proximity such that their RNA-dependent interaction can be stabilized by cross-linking.

Having established a physical interaction between eIF4G1 and Slf1, we next examined combined eIF4G and *slf1* mutant strains in terms of sensitivity to oxidative stress. A strain lacking *SLF1* was found to show similar sensitivity to oxidative stress compared with the 4G2h strain (Figure 6B). Loss of Slf1 resulted in oxidative stress sensitivity in the 4G1h strain indicating that Slf1 is required to mediate eIF4G1-dependent oxidant tolerance. Surprisingly, loss of Slf1 was found to rescue the oxidative stress sensitivity of the 4G2h strain. The reasons for this rescue are unclear but may indicate that Slf1 plays an antagonistic role in preventing eIF4G2-mediated translation of mRNAs required during oxidative stress conditions.

As a final test of the overlapping requirement for eIF4G1 and Slf1 in the translational response to oxidative stress, we compared Slf1 mRNA targets with our proteomic analysis in homogenic strains exposed to hydrogen peroxide. RNA-immune precipitation sequencing (RIP-Seq) has previously been used to identify mRNAs bound by Slf1 (64). We mapped these targets onto the volcano plot comparing the 4G1h and 4G2h proteomes exposed to oxidative stress (Figure 6C, purple highlighted circles). This analysis revealed that most of the Slf1 target mRNAs encode proteins that are increased in abundance in the 4G1h strain compared with the 4G2h strain.

### Isoform specific requirements for eIF4G during oxidative stress conditions in human cells

**Figure 7.**
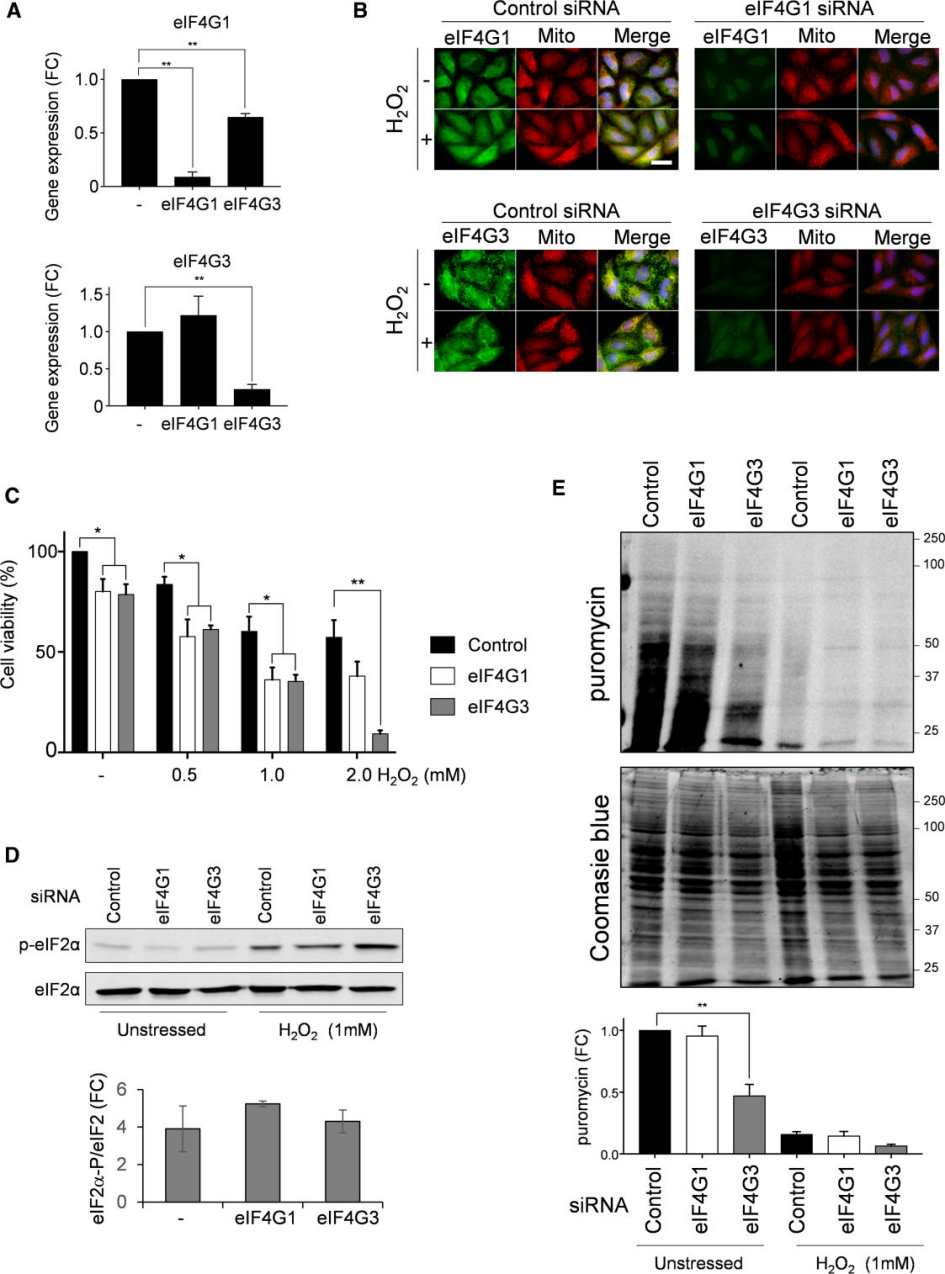
Isoform specific requirements for eIF4G in human cells during oxidative stress conditions. (**A**) qRT-PCR analyses are shown to confirm the degree of specificity for eIF4G1 and eIF4G3 knockdowns. eIF4G3 siRNA specifically lowers eIF4G3 mRNA levels without affecting eIF4G1, whereas, eIF4G1 siRNA treatment strongly reduces eIF4G1 mRNA levels and decreases eIF4G3 expression by approximately 40%. Statistical analysis was carried out using one-way ANOVA. (**B**) The indicated HeLa cells were left untreated or treated with 1 mM hydrogen peroxide for one hour. eIF4G1 and eIF4G3 were visualized by immunofluorescence staining using specific antibodies (green). Mitochondria were visualized using MitoTracker Red and merged images are shown. (**C**) HeLa cells were treated with the indicated concentrations of hydrogen peroxide and viability measured using crystal violet. Statistical analysis was carried out using one-way ANOVA to compare the viability of the control and knock-down cells. (**D**) HeLa cells were left untreated or treated with 1 mM hydrogen peroxide for one hour. Protein extracts were immunoblotted for phosphorylated eIF2α (p-eIF2α) or eIF2α. Quantification is shown from triplicate experiments comparing the fold change (FC) increase in phosphorylation in response to hydrogen peroxide exposure. (**E**) Protein synthesis was analysed using puromycin labelling under the same conditions as shown in panel D. The incorporation of puromycin into newly synthesised protein was assessed by immunoblotting. Quantification is shown from triplicate experiments normalised against the intensity of Coomassie blue staining presented as fold change (FC) in puromycin labelling.

The two eIF4G genes in *S. cerevisiae* almost certainly arose as a result of a genome duplication event in the *Saccharomyces* lineage (65), and these genes have likely been maintained to provide specific functional properties. Multiple eIF4G isoforms are also prevalent across other eukaryotic organisms, either by virtue of multiple genes or alternative splicing. To assess whether differences in eIF4G function are related to oxidative stress tolerance in other eukaryotic systems, eIF4G1 and eIF4G3 expression was specifically decreased in HeLa cells using siRNAs. qRT-PCR was used to confirm the degree of specificity to the knockdowns. eIF4G3 siRNA specifically lowered eIF4G3 mRNA levels without affecting eIF4G1 (Figure 7A). Whilst eIF4G1 siRNA treatment did strongly reduce eIF4G1 mRNA levels, it also decreased eIF4G3 expression by approximately 40%. Immunofluorescence microscopy was performed using eIF4G1 or eIF4G3 antibodies to examine eIF4G protein expression. The analysis confirmed that eIF4G1 and eIF4G3 siRNAs depleted cellular protein concentrations of eIF4G1 and eIF4G3, respectively (Figure 7B).

Treatment of HeLa cells with hydrogen peroxide causes a dose-dependent decrease in cell viability (Figure 7C). This effect on cell viability was exacerbated following eIF4G1 or eIF4G3 knockdown. Intriguingly, at the very highest hydrogen peroxide concentration tested, knockdown of eIF4G3 resulted in strong sensitivity to oxidative stress compared with the eIF4G1 knockdown suggesting isoform-specific requirements for the human eIF4Gs in promoting oxidative stress tolerance.

We confirmed that the depletion of eIF4G did not affect the basal or induced level of eIF2α phosphorylation in response to peroxide treatment of human cells suggesting that global translation is similarly regulated (Figure 7D). We used a puromycin incorporation assay to determine how the knockdowns and changes in eIF2α phosphorylation levels compare with translational inhibition (Figure 7E). Interestingly, we found that translation is inhibited following knockdown of eIF4G3 which may suggest that eIF4G3 may play a more predominant role in translation initiation during unstressed conditions than eIF4G1, similar to the more dominant role for yeast eIF4G1 compared with yeast eIF4G2. Following oxidative stress, translation was similarly inhibited under control conditions or following eIF4G1 or eIF4G3 knockdown (Figure 7E). Overall, these data suggest that in an analogous manner to the roles of yeast eIF4G1 and eIF4G2 in facilitating tolerance to oxidative stress, the human eIF4G genes might similarly differ in their capacity to specify the translational activity of specific mRNAs required for adaptation to stress.

## DISCUSSION

Many eIF4G isoforms have been described both at the level of gene duplication and alternative splicing in diverse eukaryotic organisms. For instance, *Xenopus, Arabidopsis, Drosophila* and humans all have two or more eIF4G genes, and in extreme scenarios some plants such as Soya bean contain up to eight eIF4G and eIFiso4G genes (66). This evolutionary reoccurrence of multiple distinct forms of eIF4G raises the possibility that they may have isoform-specific functions. Additionally, differences in splicing isoforms, expression levels, and tissue specific expression further contribute to the idea that different eIF4G isoforms provide an important regulatory step in translation (67,68). In this current study we found that the two isoforms of full-length eIF4G present in yeast and human cells are differentially required to mediate tolerance to oxidative stress. Understanding the functional specificity of eIF4G isoforms is important since potential disease-causing variants of eIF4G have been linked with Parkinson's disease and defects in eIF4G are associated with cancer and ineffective cancer treatments (69–71).

Yeast strains lacking eIF4G1 and human cells with reduced expression of eIF4G3 were found to be sensitive to hydrogen peroxide suggesting isoform-specificity in the translational response to oxidative stress conditions. In response to external stimuli such as ROS, global translation initiation is normally reduced, matched with complementary mechanisms that allow certain mRNAs to escape global repression (2). One key mechanism that inhibits translation initiation in eukaryotic cells is through phosphorylation of the alpha subunit of translation factor eIF2 (72). We found that eIF2α was similarly phosphorylated in response to hydrogen peroxide in yeast strains containing eIF4G1 or eIF4G2 as the sole eIF4G present or in human cells with reduced expression of eIF4G1 or eIF4G3. This indicates that the regulation of translation initiation via eIF2α phosphorylation is unaffected by the presence of different eIF4G isoforms. It is unclear how the mRNAs which show increased translational activity during oxidative stress conditions are resistant to the translation inhibition mediated via eIF2α phosphorylation and reduced ternary complex levels. Our data indicate a convergent evolutionarily advantage to eIF4G isoforms in promoting oxidative stress tolerance, suggesting that eIF4G contributes to the continued translation of key mRNAs during conditions that globally inhibit translation initiation.

Previous studies have shown that the proteome is significantly altered following exposure to ROS, independent of any changes in transcription (56,73). While the majority of protein synthesis is inhibited following oxidative stress, it is vital for the survival of the cell that the proteins involved in the response to oxidative stress continue to be made (56,73). The mechanism by which specific mRNAs are translated in response to stress is not yet fully elucidated but our quantitative proteomics data indicate that yeast eIF4G1 accounts for some of this oxidative stress dependent translation. Polysome profiling performed under high magnesium concentrations showed that mRNA-bound ribosomes increase compared with free ribosomes in eIF4G1, but not eIF4G2 containing strains, in response to oxidative stress. This altered pattern of free and mRNA-bound ribosomes in the homogenic strains was also mirrored by the patterns of eIF4G1 and eIF4G2 association across polysome gradients. eIF4G2 shifts from the heavily translated region on polysome profiles to be more associated with inactive ribosomes following oxidative stress conditions. Conversely, eIF4G1 remained associated with heavy polysomes during oxidative stress suggesting that new initiation and continued translation specifically requires eF4G1 function during oxidative stress conditions.

Further analysis aimed at revealing possible mechanistic reasons for the differences in eIF4G1 and eIF4G2 function using immunoprecipitation of the eIF4E cap binding protein did not reveal any alterations in its association with eIF4G1 or eIF4G2 following oxidative stress conditions. Similarly, cap affinity chromatography showed that different eIF4F complexes associated with eIF4G1 or eIF4G2 do not appear to be recruited to mRNAs following oxidative stress to promote translation in an isoform-specific manner. One possibility to explain the different functions of eIF4G1 and eIF4G2 is that they interact with mRNAs via different RNA binding proteins. One key difference observed in eIF4G isoform protein interactions was the specific interaction of eIF4G1 with Slf1. Slf1 is a member of the conserved La motif containing RNA-binding (LARP) proteins, and has been shown to have an important role in the oxidative stress response by binding to mRNAs specifically encoding oxidative stress response proteins (64). Slf1 remains associated with ribosomes following oxidative stress conditions and has therefore been proposed to mediate the translational response to oxidative stress via mRNA-specific translational control (64). We found that eIF4G1, but not eIF4G2, interacts with Slf1 in both an RNA-dependent and independent manner, suggesting that yeast eIF4G isoforms have different protein binding partners. This is perhaps not surprising, since although eIF4G1 and eIF4G2 are similar proteins, they also have large regions with low homology including their N-terminal regions which are not very well conserved (30).

Combined with the finding that eIF4G1 can facilitate the oxidative stress response, one possibility is that Slf1 plays a role in mediating the translation of eIF4G1-bound mRNAs. RBPs such as LARPs have increasingly been recognized as playing key roles in providing specificity to specialized ribosomes that play regulatory functions in the selective translation of subsets of mRNAs (74). Slf1 is an atypical LARP that has a conserved La-motif (LaM) RNA binding domain but lacks any RNA recognition motif (RRM) typical of the LARP family (75). Slf1 has been shown to bind small ribosomal subunits via a novel binding motif within the N-terminal region of Slf1 (64). Slf1 may therefore act as an adapter protein between specific oxidative stress related mRNAs via its LaM domain and ribosomes via its ribosome binding domain. Our data suggests these mRNAs are only accessible to eIF4G1 rather than eIF4G2. It is also interesting that the simultaneous loss of both eF4G1 and Slf1 promotes oxidant tolerance suggesting that eIF4G2 is capable of driving oxidative stress-specific translation under certain conditions. One possibility is that Slf1 acts to preclude eIF4G2 mRNA binding during oxidative stress conditions. More studies will be required to determine the functional requirement for eIF4G2 and whether it is similarly required for mRNA-specific translation during particular growth or stress conditions.

## Supplementary Material

gkad568_Supplemental_Files

## Data Availability

Proteomics data are available via ProteomeXchange with identifier PXD041682.

## Notes

Present address: Aristeidis P. Sfakianos, MRC Toxicology Unit, University of Cambridge, Tennis Court Road, Cambridge CB2 1QR, UK.

Present address: Paraskevi Kritsiligkou, Division of Redox Regulation, German Cancer Research Center (DKFZ), Im Neuenheimer Feld 280, 69120 Heidelberg, Germany.

Present address: Christopher J. Kershaw, Northwest Genomics Laboratory Hub (Manchester), Manchester Centre for Genomic Medicine, St. Mary's Hospital, Oxford Road, Manchester M13 9WL, UK.
