# Supplementary material for: Paralogous translation factors target distinct mRNAs to differentially regulate tolerance to oxidative stress in yeast
Source: Nucleic Acids Res. 2023 Jul 14;51(16):8820–35. doi: 10.1093/nar/gkad568 (PMC10484682; doi:10.1093/nar/gkad568)
Supplement: gkad568_Supplemental_Files [file gkad568_supplemental_files.zip › SupFig. 1.pdf]

Sup Figure 1

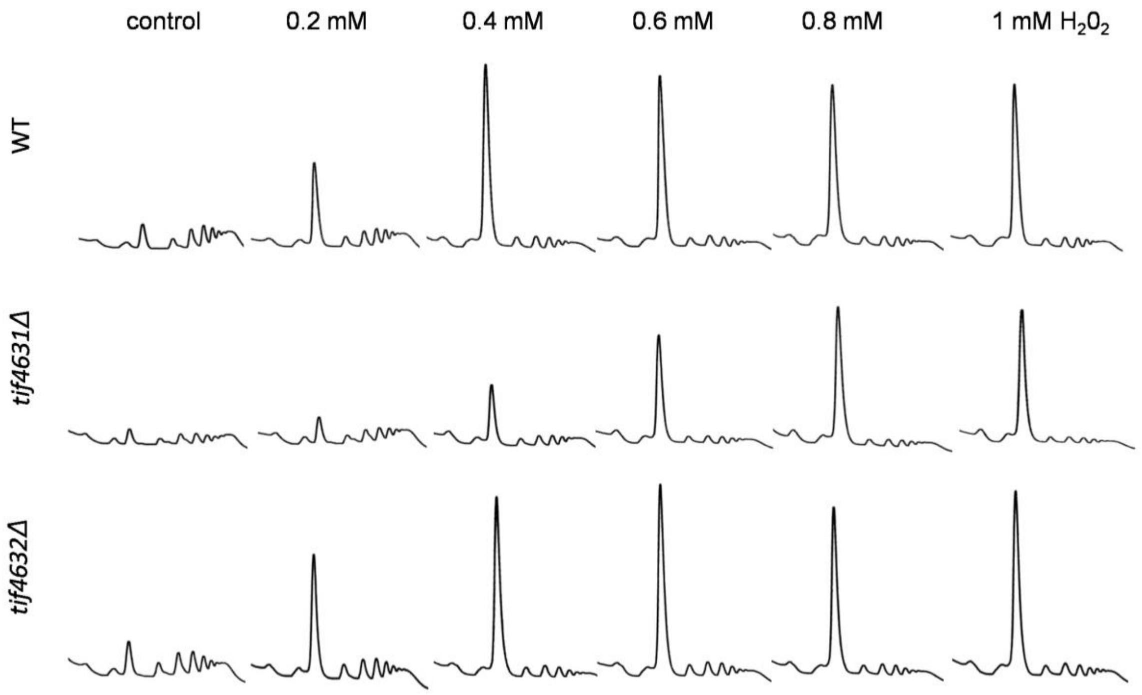

Supplementary Fig. 1. Analysis of translational activity in strains lacking eIF4G1 or eIF4G2. Representative polyribosome traces are shown for the wild-type and eIF4G deletion strains before or after treatment with the indicated concentrations of hydrogen peroxide for 15 minutes.
