# Supplementary material for: Paralogous translation factors target distinct mRNAs to differentially regulate tolerance to oxidative stress in yeast
Source: Nucleic Acids Res. 2023 Jul 14;51(16):8820–35. doi: 10.1093/nar/gkad568 (PMC10484682; doi:10.1093/nar/gkad568)
Supplement: gkad568_Supplemental_Files [file gkad568_supplemental_files.zip › Supplementary Table 1 Legend.docx]

**Supplementary Table. 1. List of proteins that show significant changes in abundance during oxidative stress conditions.** Columns A-F list proteins which were significantly (P<0.05) increased (Up) or decreased (Down) in abundance for pairwise comparisons of strains in the presence or absence of oxidative stress. Columns G-L list proteins which were significantly increased or decreased in abundance for pairwise comparisons of strains in presences of hydrogen peroxide. The listed proteins are indicated on the Volcano plots shown in Fig. 2.
